# Acute Pancreatitis Complicating L-asparaginase Therapy in Pediatric Oncology: A Case Report

**DOI:** 10.7759/cureus.111138

**Published:** 2026-06-19

**Authors:** Loubna Larda, Karima Ryouni, Elouassifi Kawtar, El Alaoui Mounia, Noufissa Benajiba

**Affiliations:** 1 Pediatrics, Abderrahim Harouchi Mother-Child Hospital, Centre Hospitalier Universitaire Ibn Rochd, Casablanca, MAR; 2 Pediatric Hematology and Oncology, Abderrahim Harouchi Mother-Child Hospital, Centre Hospitalier Universitaire Ibn Rochd, Casablanca, MAR; 3 Laboratory of Clinical Immunology, Inflammation and Allergy (LICIA), Faculty of Medicine and Pharmacy, Hassan II University, Casablanca, MAR

**Keywords:** acute lymphoblastic leukemia, acute pancreatitis, asparaginase, pediatrics, toxicity

## Abstract

Acute pancreatitis is a recognized toxicity of L-asparaginase therapy during the treatment of pediatric acute lymphoblastic leukemia (ALL). Its management requires a delicate balance between avoiding leukemic relapse and preventing pancreatic recurrence. We report the case of a five-year-and-eight-month-old girl with Philadelphia chromosome-positive B-cell ALL, initially treated with imatinib combined with the high-risk MARALL 2006 protocol, and subsequently with the COOPRALL 97 protocol following an isolated central nervous system relapse. Seventy-two hours after the 18th cumulative administration of L-asparaginase, she developed epigastric pain and vomiting. Serum lipase was greater than three times the upper limit of normal, confirming the diagnosis of acute pancreatitis. The episode was classified as grade 2 according to the Ponte di Legno group classification. After clinical and biological resolution of pancreatitis, L-asparaginase was reintroduced because of the patient’s high-risk relapsed leukemia. After 10 weeks of follow-up after reintroduction, no recurrence was observed. This case supports the feasibility of L-asparaginase rechallenge in selected high-risk pediatric patients following a multidisciplinary risk-benefit assessment.

## Introduction

Acute lymphoblastic leukemia (ALL) is the most common hematologic malignancy in children, representing approximately 25% of all pediatric cancers and 75% of all childhood leukemias [1]. The introduction of asparaginase into treatment protocols has significantly increased event-free survival rates [1]. However, this treatment is associated with several toxicities, including acute pancreatitis, which occurs in approximately 2% to 18% of children treated for ALL, depending on age, asparaginase formulation, treatment intensity, and diagnostic criteria. Although most cases resolve with supportive care and treatment interruption, asparaginase-associated pancreatitis (AAP) may be severe and can lead to pancreatic pseudocyst, necrosis, insulin requirement, respiratory failure, prolonged hospitalization, or death [2-4].

Definitive discontinuation of asparaginase may increase the risk of leukemic relapse [2-4], while its reintroduction exposes the patient to a risk of pancreatic recurrence. Therefore, reintroduction should not be systematic but may be considered in selected patients after complete clinical and biological recovery, absence of major pancreatic complications, and individualized multidisciplinary assessment of the expected oncological benefit versus the risk of pancreatic recurrence.

Here, we report a case of acute pancreatitis occurring during L-asparaginase therapy in a patient treated for B-cell ALL (B-ALL) and discuss the therapeutic management in light of literature data.

## Case presentation

A five-year-and-eight-month-old girl with no significant medical history was followed for Philadelphia chromosome-positive B-ALL (Ph+ B-ALL). She was initially treated according to the high-risk MARALL 2006 protocol in combination with imatinib, followed by the COOPRALL 97 group 2 protocol after an isolated central nervous system relapse. L-asparaginase was omitted during the second intensification phase due to the unavailability of the drug in our unit.

After 18 cumulative administrations of L-asparaginase (15 × 6,000 IU/m² in the MARALL protocol and 3 × 10,000 IU/m² in the COOPRALL protocol, totaling 120,000 IU/m²), she presented with epigastric pain, vomiting, fever, anorexia, and mucositis during post-chemotherapy febrile neutropenia. Laboratory investigations showed an elevated serum lipase level of 194 IU/L. The upper limit of normal (ULN) for serum lipase in our laboratory was 31 IU/L; therefore, the lipase level was more than six times the ULN. Serum amylase was normal (65 IU/L). Abdominal ultrasonography was performed during the episode and showed steatotic hepatomegaly, probably related to chemotherapy, without evidence of pancreatic necrosis, peripancreatic fluid collection, or other severe local pancreatic complications.

The diagnosis of acute pancreatitis was established according to accepted pediatric diagnostic criteria, requiring at least two of the following three features: compatible abdominal pain, serum amylase and/or lipase levels at least three times the ULN, and imaging findings consistent with pancreatitis [5,6]. In our patient, the diagnosis was supported by typical epigastric abdominal pain with vomiting and a serum lipase level of 194 IU/L, corresponding to more than six times the ULN in our laboratory. Therefore, imaging confirmation was not required to establish the diagnosis, although abdominal ultrasonography was performed to assess for local pancreatic complications. According to the Ponte di Legno Toxicity Working Group classification [7,8], the episode was classified as grade 2 because of the persistence of elevated pancreatic enzymes beyond 72 hours.

Management included bowel rest, intravenous rehydration, analgesia, and treatment of febrile neutropenia. Clinical improvement was observed, with discharge on day 14 and normalization of lipase levels within 24 days. After complete clinical recovery and normalization of serum lipase, the pediatric oncology team performed an individualized risk-benefit assessment. Rechallenge was considered because of the patient’s high-risk relapsed Ph+ B-ALL, the expected antileukemic benefit of continuing asparaginase therapy, the absence of major pancreatic complications, and the limited number of therapeutic alternatives. L-asparaginase was therefore reintroduced under close clinical and laboratory monitoring. No recurrence of pancreatitis was observed during the available 10-week follow-up period after reintroduction (Table 1).

**Table 1:** Clinical timeline and management of L-asparaginase-associated acute pancreatitis. ULN for serum lipase in our laboratory was 31 IU/L. A serum lipase level of 194 IU/L corresponded to more than six times the ULN. Serum amylase: 65 IU/L, within the normal range (reference range: 22–80 IU/L). ALL = acute lymphoblastic leukemia; ULN = upper limit of normal

Date	Phase/Protocol	L-asparaginase exposure	Clinical event	Laboratory/Imaging data	Decision
10/17/2023	Initial diagnosis	Start of MARALL 2006 + imatinib	Ph+ B-ALL	—	Treatment initiated
07/21/2025	Day 1, 11th reintroduction	Protocol change	Isolated central nervous system relapse	—	Switch to COOPRALL 97
08/12/2025	Day 18, VANDA	Last dose administered	—	—	—
08/15/2025	Post-asparaginase	72 hours after the last dose	Epigastric pain, vomiting, fever, anorexia	Lipase 194 IU/L, amylase 65 IU/L (normal), ultrasound without signs of severe pancreatitis	Acute pancreatitis diagnosis
08/15/2025–08/28/2025	Hospitalization	L-asparaginase discontinuation	Symptomatic management	Lipase trend: 107 → 115 → 200 → 149 IU/L, amylase: 75 IU/L	Favorable clinical course
09/07/2025	Resumption of chemotherapy	Transient L-asparaginase cessation	No new pancreatic symptoms	Lipase: 20 IU/L, amylase: 44 IU/L	Continue treatment without asparaginase
02/09/2026	Second cycle B3	L-asparaginase reintroduction	No immediate recurrence	Clinical and laboratory monitoring	Treatment continued with asparaginase
04/23/2026	Last follow-up (10 weeks)	—	No recurrence of pancreatitis	—	Favorable outcome

## Discussion

Asparaginase remains a cornerstone of pediatric ALL treatment because it depletes circulating L-asparagine, an amino acid required for leukemic lymphoblast survival. This mechanism is essential for antileukemic efficacy but may also contribute to pancreatic toxicity during treatment [9-11].

AAP is a well-recognized complication in children treated for ALL, with a reported incidence ranging from 2% to 18% depending on treatment protocols, asparaginase formulation, patient age, and diagnostic criteria [11-13]. Although most cases resolve with supportive care and treatment interruption, AAP may be severe and can lead to pancreatic necrosis, pseudocyst formation, insulin requirement, respiratory failure, prolonged hospitalization, or death [7,11-13]. Reported risk factors include older age, treatment intensity, genetic susceptibility, hypertriglyceridemia, concomitant corticosteroid use, and possibly asparaginase formulation [7,11-15]. However, prediction remains difficult, and pancreatitis may occur even in patients without classical risk factors.

In our case, pancreatitis occurred in a five-year-and-eight-month-old child after 18 administrations of native L-asparaginase, corresponding to a cumulative dose of 120,000 IU/m². This age is below the threshold most commonly associated with increased risk in several series, highlighting the role of individual susceptibility. The literature remains discordant regarding the exact contribution of the cumulative dose. Some studies suggest that AAP is not strictly dose-dependent or cumulative [16], whereas larger cohorts have reported increased risk beyond specific exposure thresholds [12]. In our patient, the intensity of the chemotherapy protocol, repeated exposure, concomitant treatment context, and individual vulnerability may have contributed to the occurrence of pancreatitis.

The main therapeutic challenge after AAP is whether asparaginase should be permanently discontinued or cautiously reintroduced. Definitive discontinuation may reduce antileukemic efficacy and increase the risk of relapse, particularly in high-risk ALL [3,4]. Conversely, re-exposure carries a substantial risk of recurrent pancreatitis, estimated in previous studies at approximately 45% to 62.5% [14,17-19]. The Ponte di Legno Toxicity Working Group recommends that the severity of the first episode should be considered when discussing re-exposure [7,8]. Therefore, rechallenge should not be systematic and must be based on individualized risk-benefit assessment.

Our case is consistent with this cautious approach. Although the episode was classified as grade 2 because of persistent enzyme elevation beyond 72 hours, the patient had no pancreatic necrosis, pseudocyst, peripancreatic collection, or organ failure. Abdominal symptoms resolved completely, and serum lipase normalized before reintroduction. These elements, together with the patient’s high-risk relapsed Ph+ B-ALL, central nervous system relapse, and limited therapeutic alternatives, supported the decision to resume L-asparaginase under close monitoring. Compared with patients who develop severe pancreatitis or major local complications, patients with grade 2 AAP without necrosis, pseudocyst, or organ failure may represent a subgroup in whom rechallenge can be discussed when the expected oncological benefit is substantial [7,8,14,17-19].

Several strategies have been proposed to reduce the risk of recurrence after rechallenge, including switching from native L-asparaginase to peg-asparaginase or Erwinia-derived asparaginase. However, available evidence does not clearly demonstrate that changing formulation eliminates the risk of recurrence, suggesting that pancreatic toxicity is probably related to a class effect rather than to a single formulation [11,17,20]. In this context, careful post-rechallenge monitoring remains essential and should include systematic clinical assessment for abdominal pain or vomiting, serial pancreatic enzyme measurements, and imaging when symptoms or enzyme elevation suggest recurrence.

No recurrence was observed in our patient during the available 10-week follow-up period after L-asparaginase reintroduction. However, this favorable short-term outcome should be interpreted cautiously. The follow-up period was relatively limited and is insufficient to draw firm conclusions regarding the long-term safety of rechallenge or the risk of late pancreatic complications. Moreover, this is a single case report, and the outcome cannot be generalized to all patients with AAP. Larger studies and local case series are needed to better define which patients may safely benefit from asparaginase re-exposure after pancreatitis.

This case highlights the importance of early suspicion of acute pancreatitis in any child receiving asparaginase who develops abdominal pain or vomiting. It also illustrates that, in selected very high-risk hematological situations, L-asparaginase rechallenge may be considered after complete clinical and biological recovery, absence of major pancreatic complications, and multidisciplinary risk-benefit assessment.

## Conclusions

AAP is a severe toxicity in pediatric oncology and may compromise the continuation of optimal antileukemic therapy. Therapeutic decisions must balance the risk of pancreatic recurrence against the potential loss of oncological benefit resulting from asparaginase discontinuation. In selected very high-risk hematological cases, L-asparaginase reintroduction may be considered on a case-by-case basis after complete clinical and biological recovery, absence of major pancreatic complications, and multidisciplinary risk-benefit assessment. However, this report describes a single case with a limited follow-up period after rechallenge; therefore, its findings should be interpreted with caution. Larger studies and local case series are needed to better define safe criteria for asparaginase reintroduction and to adapt management strategies to our clinical context.
